# Phloretin Attenuates *Listeria monocytogenes* Virulence Both *In vitro* and *In vivo* by Simultaneously Targeting Listeriolysin O and Sortase A

**DOI:** 10.3389/fcimb.2017.00009

**Published:** 2017-01-19

**Authors:** Jianfeng Wang, Bowen Liu, Zihao Teng, Xuan Zhou, Xiyan Wang, Bing Zhang, Gejin Lu, Xiaodi Niu, Yongjun Yang, Xuming Deng

**Affiliations:** Key Laboratory of Zoonosis, Ministry of Education, College of Veterinary Medicine, Jilin UniversityChangchun, China

**Keywords:** *Listeria monocytogenes*, listeriolysin O, sortase A, phloretin, anti-infective therapy

## Abstract

The critical roles of sortase A (SrtA) and listeriolysin O (LLO) in *Listeria monocytogenes* pathogenicity render these two virulence factors as ideal targets for the development of anti-virulence agents against *L. monocytogenes* infection. Additionally, the structures of SrtA and LLO are highly conserved among the members of sortase enzyme family and cholesterol dependent toxin family. Here, phloretin, a natural polyphenolic compound derived from apples and pears that has little anti-*L. monocytogenes* activity, was identified to simultaneously inhibit LLO expression and neutralize SrtA catalytic activity. Phloretin neutralized SrtA activity by causing a conformational change in the protein's active pocket, which prevented engagement with its substrate. Treatment with phloretin simultaneously reduced *L. monocytogenes* invasion into host cells and blocked the escape of vacuole-entrapped *L. monocytogenes* into cytoplasm. Further, *L. monocytogenes*-infected mice that received phloretin showed lower mortality, decreased bacterial burden and reduced pathological injury. Our results demonstrate that phloretin is a promising anti-infective therapeutic for infections caused by *L. monocytogenes* due to its simultaneous targeting of SrtA and LLO, which may result in fewer side effects than those caused by other antibiotics.

## Introduction

*Listeria monocytogenes*, a Gram-positive, opportunistic bacterial species, is a common foodborne intracellular pathogen and the etiological agent of listeriosis, an infection characterized by gastroenteritis, septicemia, meningitis and miscarriage in humans and animals (Dussurget et al., 2004; Posfay-Barbe and Wald, 2009). *L. monocytogenes* is ubiquitous in the natural environment, and infection with this bacterium results in high mortality, with a rate of 30% or higher, despite early intervention with antimicrobial therapy (Posfay-Barbe and Wald, 2009; Walland et al., 2015). The strong resistance of *L. monocytogenes* to extreme environments, such as those with a low pH, a high concentration of salt or a cold temperature, makes it difficult to avoid food contamination (Martinez-Suarez et al., 2016). *L. monocytogenes* can penetrate the host intestinal barrier by invading the intestinal epithelium cells, after which it can multiply within the liver and spleen, cross the fetal-placental barrier if present, and penetrate the blood-brain barrier (Cossart, 2011). These critical processes are closely associated with virulence factors for *L. monocytogenes*, suggesting that targeting these factors could serve as an alternative therapeutic strategy for bacterial infection (Dussurget et al., 2004). Unlike traditional antibiotic therapy, targeting virulence factors, which are not essential for bacterial viability, will create less selective pressure for developing drug resistance. This approach would also preserve the host's endogenous microbiome and expand the repertoire of bacterial targets (Clatworthy et al., 2007).

*L. monocytogenes* gains access to the host intracellular environment via internalin and other LPXTG-anchored cell-wall proteins, which promote the bacterium's entry into both professional and non-professional phagocytes through phagocytosis or a zipper-like mechanism (Dussurget et al., 2004; Carvalho et al., 2014). During this process, *L. monocytogenes* becomes entrapped in internalization vacuoles and then escapes into cytosol, where it can rapidly replicate by secreting a pore-forming toxin known as listeriolysin O (LLO) (Hamon et al., 2012). Another surface protein, ActA, hijacks the actin-assembly machinery of the host cell, which powers the motility of *L. monocytogenes* into or between infected cells and neighboring cells without exposing the bacteria to extracellular space, enabling it to evade the host immune system (Reddy and Lawrence, 2014). Thus, virulence factors produced in the correct host environment and at the correct time are indispensable for successfully establishing a robust infection by *L. monocytogenes*. Previous gene knockout studies have shown that disruption of any of these processes significantly attenuates the virulence of *L. monocytogenes* both *in vitro* and *in vivo* (Bierne et al., 2002; Lety et al., 2002; Yin et al., 2011). The anchoring of the LPXTG motif that is found in the surface proteins of most Gram-positive bacteria is catalyzed by the transpeptidase sortase A (SrtA) (Bierne et al., 2002). Thus, this protein is another target for anti-infective therapy. Furthermore, the virulence-associated proteins required for these processes are not essential for bacterial growth. Therefore, interruption of the bacterial lifecycle by targeting the virulence factors described above would be an alternative strategy for fighting *L. monocytogenes* infection without adding selective pressure.

In agreement with this idea, in previous works, we significantly decreased the pathogenicity of *L. monocytogenes* both *in vitro* and *in vivo* by targeting LLO or SrtA using treatments with natural compounds (Wang et al., 2015a; Li et al., 2016). We therefore reasoned that a better anti-infective therapeutic effect may be observed using an inhibitor that simultaneously targets both LLO and SrtA. Phloretin, a natural dihydrochalcone flavonoid compound found in apples and in apple-derived products, has been proven to possess a potent antioxidant activity in peroxynitrite scavenging and the inhibition of lipid peroxidation (Rezk et al., 2002). Here, phloretin was found to simultaneously inhibit LLO production and neutralize SrtA activity by using hemolysis assay and SrtA enzyme activity inhibition assay, respectively.

The invasion of *L. monocytogenes* into host cells and bacterial escape from internalization vacuoles into cytoplasm were both blocked by treatment with phloretin. Furthermore, phloretin treatment dramatically reduced *L. monocytogenes* virulence in mice. The results presented in this study indicate that phloretin is an effective anti-infective agent for *L. monocytogenes* through its simultaneous targeting of LLO and SrtA.

## Materials and methods

### Bacterial strains, reagents, and growth conditions

Phloretin was obtained from Sigma-Aldrich (St. Louis, MO, USA). The *L. monocytogenes* EGDe wild-type strain BUG 1600, *L. monocytogenes* EGDeΔ*srtA* strain BUG 1777 and *L. monocytogenes* EGDeΔ*hly* strain BUG 3649 were gifts from Dr. Pascale Cossart (Institut Pasteur, Paris, France). *L. monocytogenes* strains were grown in Trypticase Soy Broth (TSB, Qingdao Hope Biol-Technology Co., Ltd) supplemented with or without phloretin at 37°C.

### Hemolysis assay

Overnight cultures of EGDe were enlarged and cultured into fresh TSB (1:100) at 37°C for 2 h with shaking and then treated with various concentrations of phloretin until the optical density (OD) at 600 nm reached 2.0, at which point they were harvested (8000 g, 5 min). The bacterial culture supernatants were incubated with rabbit erythrocytes (final concentration of 2.5%) in PBS (35 mM Na_3_PO4, 125 mM NaCl, 0.5 mg/mL BSA, pH 5.5) at 37°C for 30 min, and then the hemolytic activity of each sample was determined by measuring the release of hemoglobin in supernatants at an OD of 543 nm after centrifugation (8000 g, 1 min).

To examine the direct effect of phloretin on hemolysis induced by bacterial culture supernatants, the hemolytic activities of bacterial culture supernatants pre-incubated with various concentrations of phloretin were determined as described above.

### Western blotting assay

Equal volume of the supernatants described as in hemolysis assay was separated by SDS-PAGE, and proteins were transferred onto polyvinylidene fluoride membranes. After blocking in 5% non-fat dry milk, the membranes were incubated with a primary rabbit anti-LLO antibody (Abcam) diluted 1:2000 and a horseradish peroxidase-conjugated secondary antibody (Proteintech) diluted 1:3000. Signals were visualized on a Tanon-4200 imager using Amersham ECL Western blotting detection reagents (GE Healthcare, Buckinghamshire, UK).

To detect LLO synthesis in *L. monocytogenes*, the above-described bacterial pellets were lysed by sonication in PBS (35 mM Na_3_PO4, 125 mM NaCl, 0.5 mg/mL BSA, pH 5.5). The cell lysate was centrifuged at 13,000 rpm for 10 min, and the level of LLO was detected by Western blotting.

### Agar assay and β-galactosidase activity analysis

The *phly-lacZ* plasmid was a kind gift from Dr. Birgitte Kallipolitis (University of Southern Denmark) and was electroporated into EGDe cells to perform an agar assay as described previously (Kastbjerg et al., 2010). A 25-ml aliquot of TSB agar was melted at 44°C and supplemented with X-gal (150 μg/ml) and kanamycin (50 μg/ml) before being thoroughly mixed with 1 ml of an overnight culture of bacteria containing the *phly-lacZ* plasmid. Nine wells were made per plate following desiccation in Class II biosafety cabinet at room temperature. A 30-μl aliquot of phloretin or benzalkonium bromide at varying concentrations was added to each well. The plates were incubated for 48 h at 37°C. The β-galactosidase activities of the *phly-lacZ*-containing bacteria treated with various concentrations of phloretin were quantitatively determined as previously described (Larsen et al., 2006).

### Construction, expression and purification of SrtA and its mutants

Expression vectors for SrtA mutants (SrtA T90A, SrtA I103A, and V129A) were constructed using a QuikChange site-directed mutagenesis kit (Stratagene, La Jolla, CA, USA) based on the SrtAΔN70-pGEX-6P-1 construct, a vector created in our lab for the expression of wild type SrtA (WT-SrtA) (Li et al., 2016). The primer pairs for these three mutations were as follows: T90A forward, 5′-GAATATACAGTTGCGGAAACAAAAAC-3′; T90A reverse, 5′-GTTTTTGTTTCCGCAACTGTATATTC-3′; I103A forward, 5′-GATGAAACAGAAGTAAGCGTTGCGGATAATACGAAAGATGCTAG-3′; I103A reverse, 5′-CTAGCATCTTTCGTATTATCCGCAACGCTTACTTCTGTTTCATC-3′; V129A forward, 5′-GAAACGTTTTGTCGCAGCGGGTGAGCTAGAAAAAAC-3′; and V129A reverse, 5′-GTTTTTTCTAGCTCACCCGCTGCGACAAAACGTTTC-3′. The expression and purification of WT-SrtA and its derivatives was carried out as described in our previous study (Li et al., 2016).

### Sortase A enzyme activity inhibition assay

WT-SrtA or its derivatives (4 mM) were incubated with the indicated concentrations of phloretin (14.59 or 58.34 μM) at 37°C for 20 min in reaction buffer (50 mM Tris-HCl, 5 mM CaCl_2_, and 150 mM NaCl, pH 8.0), and then a catalytic reaction was triggered by adding the fluorescent peptide substrate Dabcyl-QALPTTGEE (Edans) (GL Biochem, Shanghai, China). Following another incubation at 37°C for 1 h, the fluorescence intensity of each sample was determined at emission and excitation wavelengths of 350 and 520 nm, respectively, in a black 96-well plate.

### Susceptibility assays

The minimal inhibitory concentration (MIC) of phloretin for *L. monocytogenes* EGDe cells (5 × 10^5^ CFUs/ml) was determined in TSB at 37°C according to the procedure described by Langfield et al. (2004). The growth of *L. monocytogenes* EGDe cells cultured with the indicated concentrations of phloretin was tested by determining the OD of each sample at 600 nm every 30 min.

### Molecular modeling

The starting structure of SrtA for molecular dynamics (MD) simulations was constructed using a homology modeling method based on an X-ray crystal structure from the Protein Data Bank (PDB code 4CDB). The standard docking procedure for a rigid protein and a flexible ligand in AutoDock 4.0 was used for molecular docking. Following this, molecular modeling of SrtA with phloretin was performed using computational biology methods described in our previous reports (Dong et al., 2013; Niu et al., 2013).

### Determination of phloretin binding affinity for SrtA

The binding constant (KA) for phloretin with SrtA was measured using a fluorescence-quenching method. A 280-nm excitation wavelength with a 5-nm band-pass and a 345-nm emission wavelength with a 10-nm band-pass were used for the measurements. The details of the protocol used to perform the measurements were as previously described (Bandyopadhyay et al., 2002; Jurasekova et al., 2009).

### Cell culture

The Caco-2 cell line was maintained in RPMI-1640 medium with 10% fetal bovine serum, non-essential amino acids and antibiotics in a humidified atmosphere at 37°C with 5% CO_2_. The cells were routinely adjusted to a concentration of 3 × 10^5^ cells per ml, seeded into 24-well plastic culture microplates (3 × 10^5^ per microplate) and cultured for 48 h for immunostaining and the determination of intracellular bacterial growth. To assay LDH release, cell monolayers were incubated overnight in culture medium without antibiotics in 96-well plates (2 × 10^4^ cells per well).

### LDH release assay

Caco-2 cells grown in 96-well microplates were infected with *L. monocytogenes* pre-exposed or not to phloretin at a multiplicity of infection (MOI) of 100. Following infections with discriminatory concentrations of phloretin for 5 h, the LDH released into the supernatants of the co-culture system was determined using a Cytotoxicity Detection Kit (LDH; Roche, Basel, Switzerland) as previously described (Wang et al., 2015a).

### Determination of intracellular viable bacteria

Caco-2 cells were co-cultured with *L. monocytogenes* at an MOI of 100. Following incubation with or without phloretin (58.34 μM) for 1, 3, or 5 h, intracellular viable bacteria were counted using a colony-forming unit (CFU) assay after thoroughly lysing the cells with 1% bioclean Triton-X100 as previously described (Decatur and Portnoy, 2000). In each case, gentamicin (20 μg/ml) was added 30 min after bacterial internalization.

### Immunostaining and confocal microscopy

Caco-2 cells were inoculated with bacterial suspensions pre-cultured with or without phloretin (58.34 μM) and adjusted to obtain an MOI of 100. Following incubation with or without phloretin for 0.5 h, the cells were washed three times with PBS before treatment with gentamicin (10 μg/ml) with or without phloretin.

For invasion assays, cells were washed three times with PBS at 1 h post-infection, fixed with 4% paraformaldehyde for 20 min, blocked with 5% BSA in PBS for 1 h and incubated with a rabbit *L. monocytogenes*-specific antibody (Abcam) and an Alexa Fluor 594-conjugated chicken anti-rabbit antibody (Molecular Probes) to stain extracellular bacteria. A 0.3% concentration of Triton X-100 was added to permeabilize the cells for 4 min prior to stain the total bacteria (intracellular + extracellular) with a rabbit *L. monocytogenes*-specific antibody (Abcam) and an Alexa Fluor 488-conjugated chicken anti-rabbit antibody (Molecular Probes).

To measure bacterial escape from vacuoles into cytoplasm, cells were washed three times with PBS at the indicated time points, covered with 4% paraformaldehyde for 20 min to fix the cytoskeleton and adherent or intruded bacteria, permeabilized with 0.3% Triton X-100 for 4 min and blocked with 5% BSA in PBS for 1 h. F-actin in host cells and bacteria was stained with Alexa Fluor 488-conjugated antibodies against phalloidin (green) and a rabbit *L. monocytogenes*-specific antibody (Abcam) conjugated to Alexa Fluor 594 (red), respectively; nuclei were stained in blue with DAPI.

### Mouse infections

Female 6- to 8-week-old BALB/c mice were purchased from Liao Ning Chang Sheng Biotechnology Co., LTD. The mice used for the experiments were housed and handled in accordance with the guidelines established by the United States National Institutes of Health. These studies were reviewed and approved by the Institutional Animal Care and Use Committee of Jilin University.

Overnight cultures of *L. monocytogenes* were enlarged by culturing into fresh TSB (1:100) at 37°C with shaking until the OD at 600 nm reached 1.0, at which point they were harvested. The cell pellets were washed and suspended in normal saline. To establish an infection model, 200 μL prepared bacterial suspension (1 × 10^8^ CFU/ml) was intraperitoneally inoculated into BALB/c mice. The infected mice in the treatment group were subcutaneously administered 150 mg/kg of phloretin 2 h after infection and again at 8-h intervals. The infected mice in the positive group were injected with an equal volume of dimethyl sulfoxide (DMSO) at the same time points. Finally, a group of healthy mice received only phloretin to examine the toxicity of the drug. Each group had 10 mice, and the therapeutic trial lasted for 96 h; survival statistics were collected throughout this period.

For histopathologic analysis, the mice were euthanized with anesthesia following cervical dislocation at 48 h post-infection. Liver and spleen tissues were removed, fixed with formalin, embedded in paraffin, stained with hematoxylin and eosin and visualized by light microscopy. To analyze the bacterial burden in tissue, the liver and spleen tissues collected from the sacrificed mice were weighed, lysed in 2% Triton X-100 and inoculated onto TSB agar plates at 37°C overnight.

### Statistical analysis

All experimental data were expressed as the mean ± SEM (*n* ≥ 3). GraphPad Prism 5.0 was used for statistical analysis using Student's *t*-test. Significance levels of *p* < 0.05 and *p* < 0.01 are indicated in the figures.

## Results

### Phloretin simultaneously inhibits LLO expression and neutralizes SrtA activity in *L. monocytogenes*

**Figure 1:**
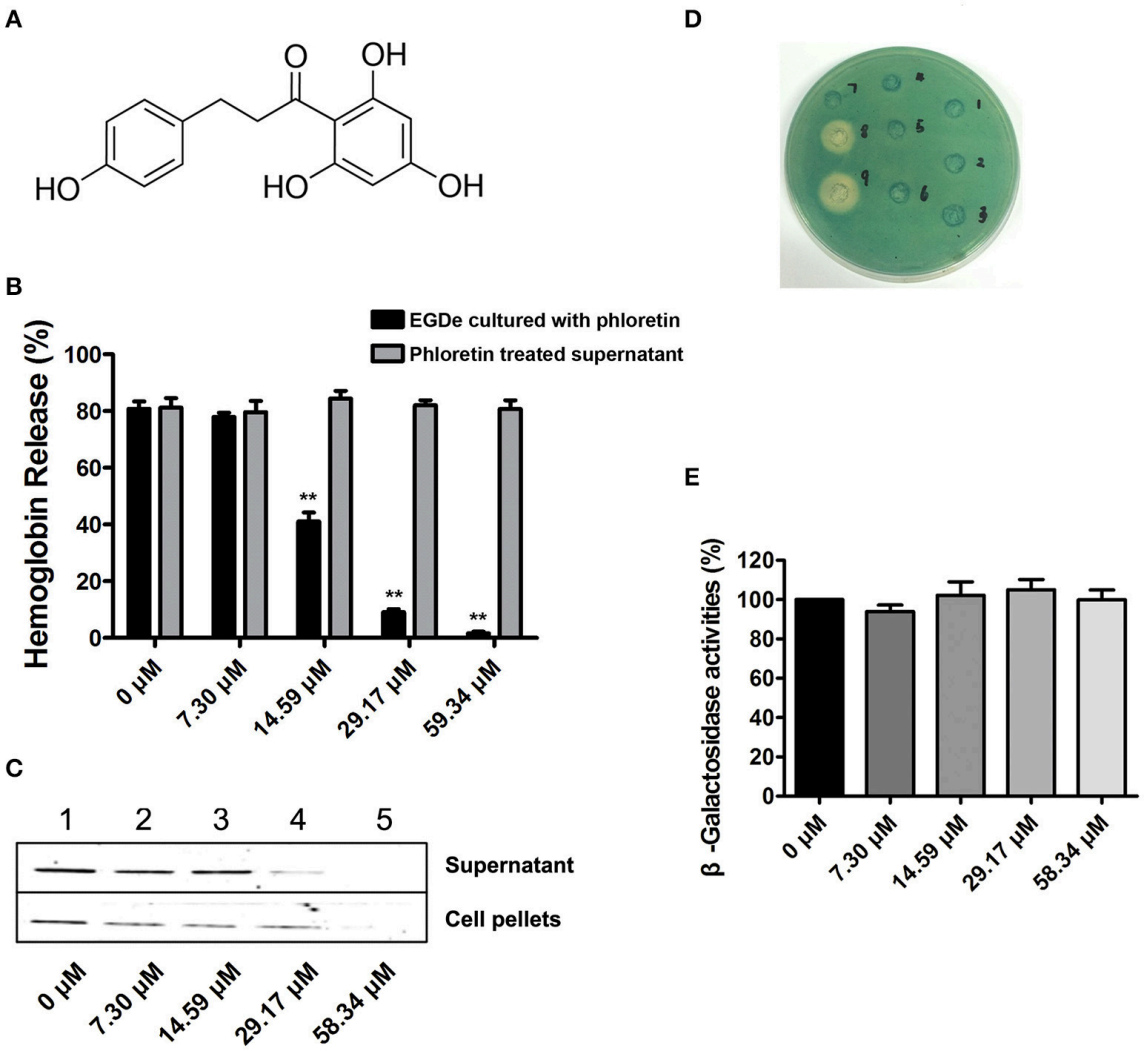
**Phloretin inhibits LLO expression in ***L. monocytogenes***. (A)** The chemical structure of phloretin. **(B)** The effect of phloretin on the hemolytic activities of bacterial culture supernatants. Inhibition of hemolytic activity in culture supernatants of *L. monocytogenes* after co-culture with phloretin. *L. monocytogenes* was co-cultured with various concentrations of phloretin until reaching a stationary phase, and the hemolytic activities of the culture supernatants from each sample were determined using hemolysis assays. However, no inhibition of hemolytic activity for bacterial culture supernatants was observed following a direct pre-incubation with various concentrations of phloretin. **(C)** Western blotting analysis to detect LLO levels in culture supernatants and cell pellets. *L. monocytogenes* was co-cultured with various concentrations of phloretin until reaching a stationary phase, and the LLO level in the culture supernatants and cell pellets were determined by immunoblotting. Lanes 1–5, samples treated with 0, 7.30, 14.59, 29.17, and 58.34 μM phloretin. **(D,E)** No inhibition of *hly* transcription was produced by phloretin. Bacteria containing *phly-lacZ* plasmids were incubated with various concentrations of phloretin in TSB agar **(D)** or TSB **(E)**. **(D)** Phloretin was added at increasing concentrations in wells 1–6 (0–116.68 μM); well 8 contained a 40% benzalkonium bromide solution; and well 9 contained a 60% benzalkonium bromide solution. **(E)** β-galactosidase activity was evaluated using a β-galactosidase staining kit and the values were obtained by comparing with data of phloretin-free samples (taken as 100%). ^*^P < 0.05; ^**^P < 0.01.

**Figure 2:**
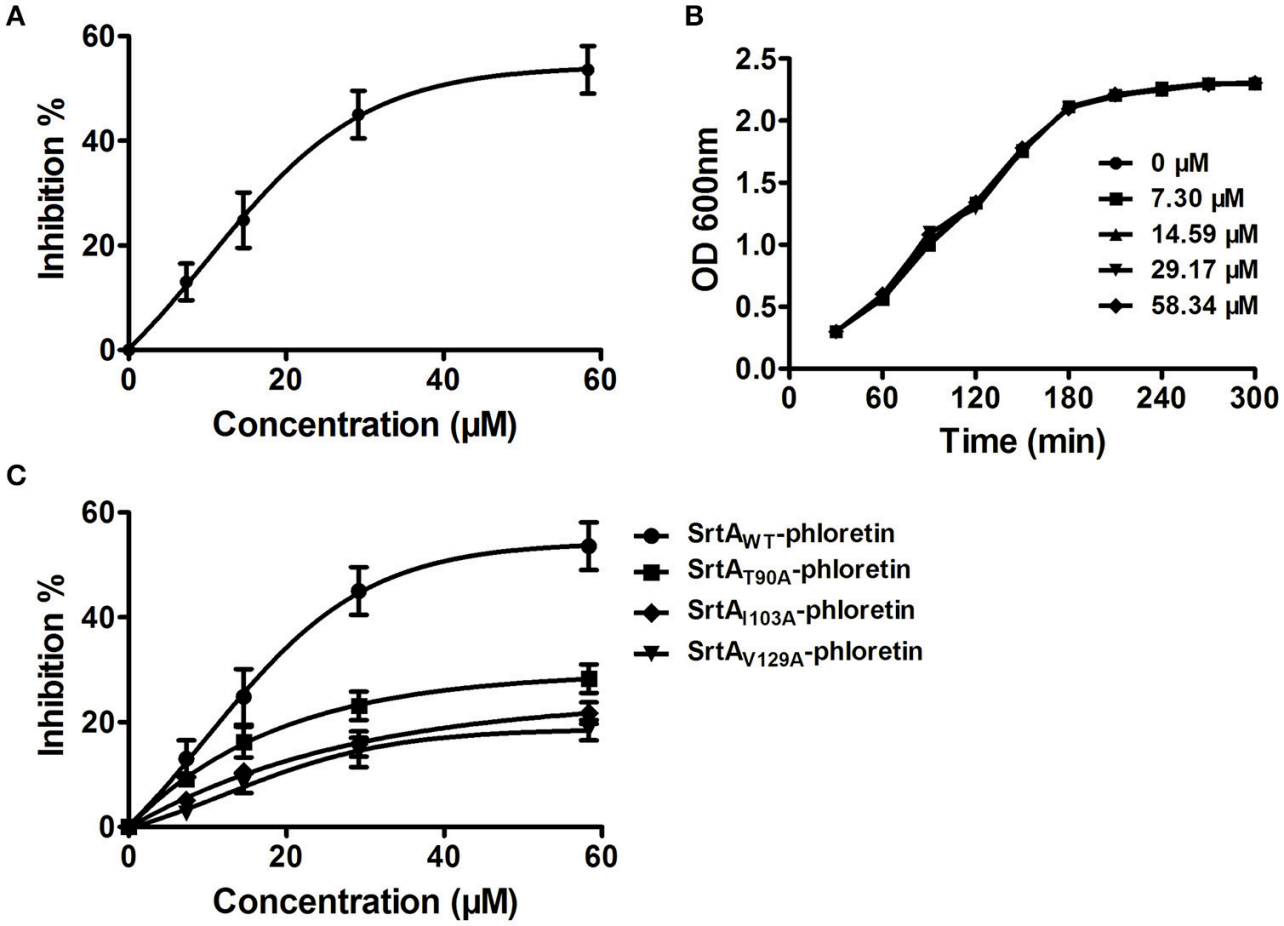
**Phloretin inhibits SrtA catalytic activity in ***L. monocytogenes***. (A)** Inhibition of SrtA catalytic activity by phloretin. Purified SrtA was pre-incubated with phloretin at 37°C for 30 min and then mixed with the fluorescent peptide substrate Dabcyl-QALPTTGEE-Edans. Following incubation at 37°C for 1 h, the fluorescence values of the reaction system were determined using emission and excitation wavelengths of 350 and 520 nm, respectively. **(B)** Growth curve of *L. monocytogenes* in the presence of various concentrations of phloretin. **(C)** The effects of phloretin on the catalytic activities of WT-SrtA and SrtA mutants.

Potential inhibitors of *L. monocytogenes* virulence factors were identified using a phenotypic assay based on protein function (Wang et al., 2015a,b; Li et al., 2016). In this work, the hemolytic activities of culture supernatants of *L. monocytogenes* EGDe cells dose-dependently decreased after co-culture with various concentrations of phloretin (7.3–58.34 μM) (Figures 1A,B). However, pre-incubation with phloretin did not affect the hemolytic activities of bacterial culture supernatants (Figure 1B), suggesting that this compound effectively inhibits LLO maybe by preventing its expression. To further validate these observations, western blotting was employed, and the results were consistent with those from the hemolytic activity assay: the LLO levels in bacterial culture supernatants and cell pellets co-cultured with phloretin were reduced in a concentration-dependent manner (Figure 1C). These data implied that the observed LLO inhibition was due to a phloretin-induced decrease of LLO synthesis at the transcriptional or translational level. However, phloretin treatment did not affect the transcription of *hly*, the gene encoding LLO, according to an agar assay (Figure 1D) and β-galactosidase activity analysis (Figure 1E) using a *phly-lacZ*-containing bacterial construct. Furthermore, phloretin also effectively inhibited the catalytic activity of SrtA based on a SrtA activity inhibition assay that used the fluorescent peptide substrate Dabcyl-QALPTTGEE-Edans. The IC_50_ for phloretin-induced inhibition was 37.24 μM under our experimental conditions (Figure 2A). Importantly, phloretin showed little anti-microbial activity against *L. monocytogenes*, with a MIC of 933.44 μM, which is 16-fold higher than the concentration used for the SrtA activity inhibition assay and hemolytic activity analysis. Additionally, the growth of *L. monocytogenes* was not apparently affected following treatment with phloretin at the concentrations required for sufficient inhibition of LLO expression and SrtA catalytic activity (Figure 2B). Taken together, our results established that phloretin, a natural polyphenolic compound widely found in apple and pears with little anti-*L. monocytogenes* activity, was an effective inhibitor that simultaneously neutralized SrtA activity and inhibited LLO expression in *L. monocytogenes*. These results indicate that phloretin may represent an effective anti-infective candidate for *L. monocytogenes* infection without putting selective pressure on this bacterium.

### Molecular dynamics simulations of the SrtA-phloretin interaction

**Figure 3:**
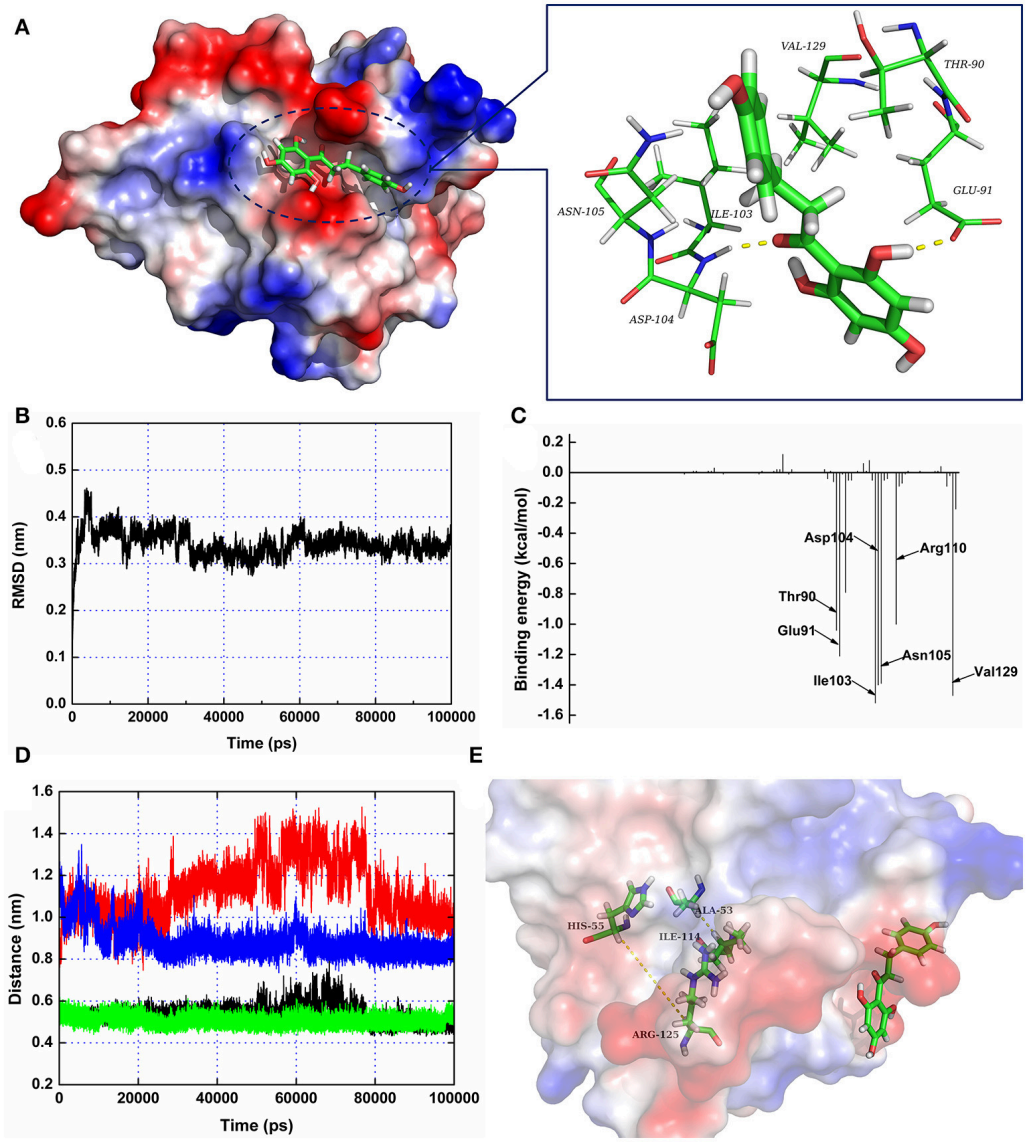
**The 3D structure of SrtA in complex with phloretin determined by molecular modeling**. **(A)** The 3D structure of the SrtA-phloretin complex. **(B)** The RMSD values of the backbone atoms of SrtA during MD simulations of the binding of SrtA to phloretin. **(C)** Decomposition of binding energy on a per-residue basis for the residues in the binding site of SrtA as the SrtA-phloretin complex formed. **(D,E)** Distances between Ala53-Ile114 and His55-Arg125 as a function of time. The black line (Ala53-Ile114 in the protein complex), red line (His55-Arg125 in the protein complex), green line (Ala53-Ile114 in free protein) and blue line (His55-Arg125 in free protein) represent the distances between the same residues.

To further characterize the mechanism underlying phloretin-induced inhibition of SrtA activity, the preferential binding mode of SrtA with phloretin was determined based on 100-ns MD simulations. The details of the interaction formed between SrtA and phloretin were obtained using molecular modeling. To indicate the convergence of a complex structure, the RMSD values for SrtA in the whole system as a function of time are shown in Figure 3B. The backbone RMSD of SrtA reached equilibrium after ~20 ns of simulation, validating that the final 60 ns of the simulation were suitable for analysis. As shown in Figure 3A, the potential binding mode of phloretin with the active site of SrtA was determined based on 100-ns MD simulations. The results revealed that phloretin can bind to SrtA via intermolecular forces. In this complex system, phloretin could localize to the specific binding pocket of SrtA, adjacent to the active region of SrtA. Furthermore, the side chains of phloretin can form strong interaction with Thr90, Glu91, Ile103, Asp104, Asn105, and Val129 in SrtA (Figure 3A).

To identify the binding sites used by the SrtA-phloretin complex system, the MM-PBSA method was used to calculate the binding free energy between the residues surrounding the binding site and phloretin. As shown in Figure 3C, Ile103, Asp104, Asn105, and Val129 have the strongest total binding energy contribution, with an ΔEtotal of ≤ −1.4 kcal/mol. In addition, residues Thr90, Glu91, and Arg110 also have an appreciable total binding energy contribution, with an ΔEtotal of ≤ −1.0 kcal/mol (Figure 3C). These results suggest that these five residues are key residues for the binding of phloretin to SrtA.

The total binding free energy for the SrtA-phloretin complex and the detailed energy contributions were calculated according to the MM-PBSA approach, as summarized in Table 1. According to the calculation results, the binding free energy, ΔGbind, of the interaction between phloretin and SrtA decreased in the following order: WT-SrtA > mutants, meaning that WT-SrtA has the strongest ability to bind with phloretin. By fluorescence-quenching spectroscopy, we measured the ΔGbind and the number of binding sites between phloretin and the two mutants; these results were highly consistent with those obtained by computational methods (Table 1). Furthermore, the inhibitory effect of phloretin against SrtA mutant activity (SrtA T90A, SrtA I103A, and V129A) is much lower than that against WT-SrtA (Figure 2C). These results indicate that the information generated by the MD simulations of the SrtA-phloretin complex is reliable. Thus, upon the binding of phloretin with the active region of SrtA (residues Thr90, Glu91, Ile103, Asp104, Asn105, Arg110, and Val129), the biological activity of SrtA was inhibited.

**Table 1:** **The binding free energies (kcal/mol) of the WT-LIG, T90A-LIG, I103A-LIG, and V129A-LIG systems based on computational methods, and the values of the binding constants (KA) based on fluorescence-quenching spectroscopy**.

	**WT-SrtA**	**T90A**	**I103A-LIG**	**V129A**
Computational method	−13.8 ± 1.9	−9.7 ± 1.4	−8.6 ± 1.7	−8.5 ± 1.2
*K*_A_ (1 × 10^4^) L·mol^−1^	7.93 ± 1.32	5.71 ± 1.44	5.80 ± 1.33	4.98 ± 1.37

### Phloretin simultaneously inhibits *L. monocytogenes* invasion and escape from internalization vacuoles in Caco-2 cells

**Figure 4:**
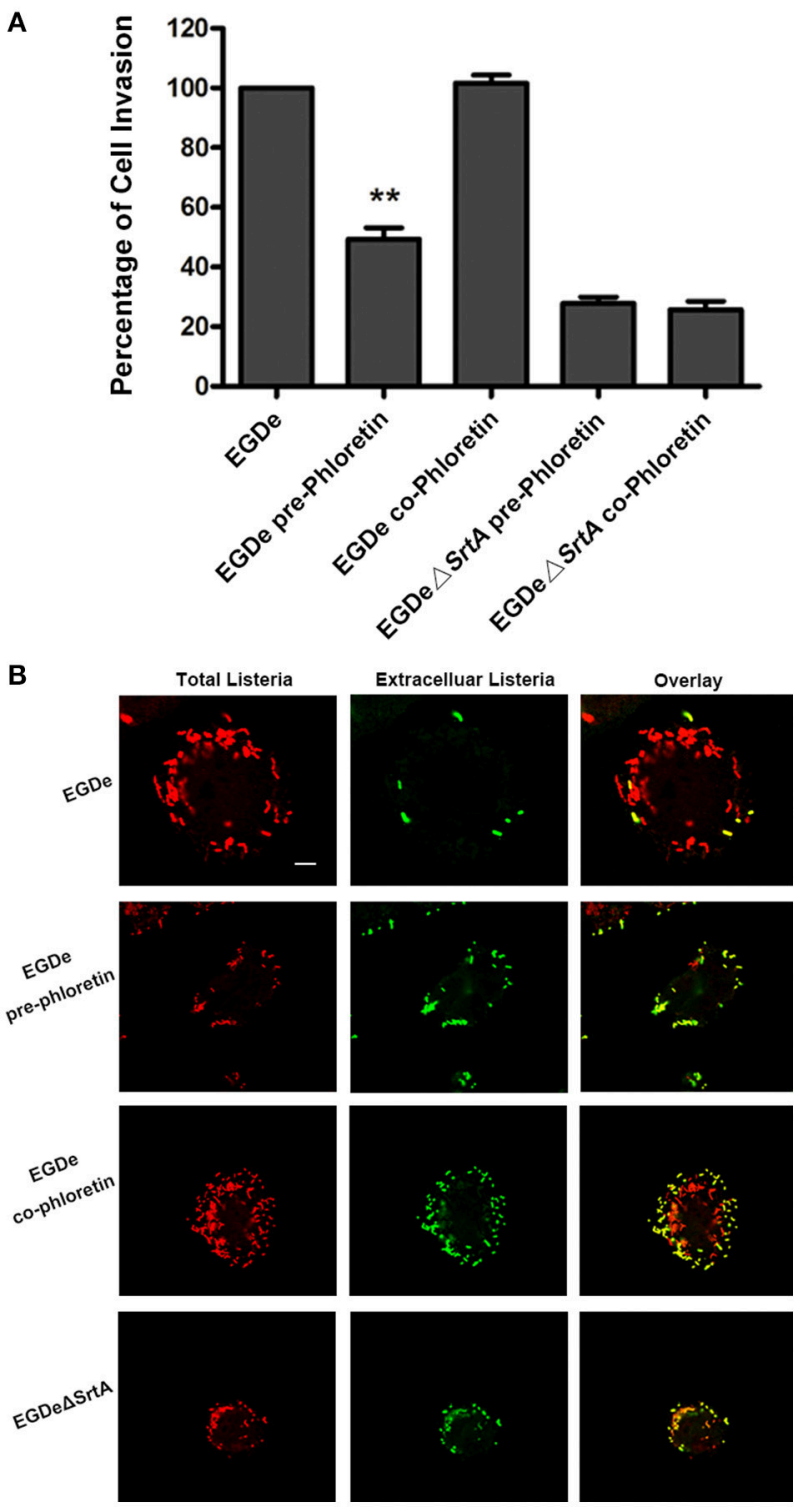
**Phloretin reduces ***L. monocytogenes*** invasion into Caco-2 cells. (A)** Caco-2 cells were infected with phloretin (58.34 μM) pre-treated *L. monocytogenes* or *L. monocytogenes* with or without phloretin treatment (58.34 μM) during infection for 1 h, and the total number of viable bacteria in cells was counted after lysing the cells. **(B)** *L. monocytogenes* pre-cultured with or without phloretin (58.34 μM) were co-incubated with host cells at an MOI of 100 in the presence or absence of phloretin (58.34 μM). At 1 h post-infection, extracellular and total bacteria were stained with Alexa594-conjugated antibodies (red) and Alexa488-conjugated antibodies (green), respectively. Consistent with the samples infected with the EGDeΔ*srtA* strain, only the cells infected with phloretin-pre-treated *L. monocytogenes* showed reduced bacterial entry. Scale bar, 10 μm. ^*^P < 0.05; ^**^P < 0.01.

**Figure 5:**
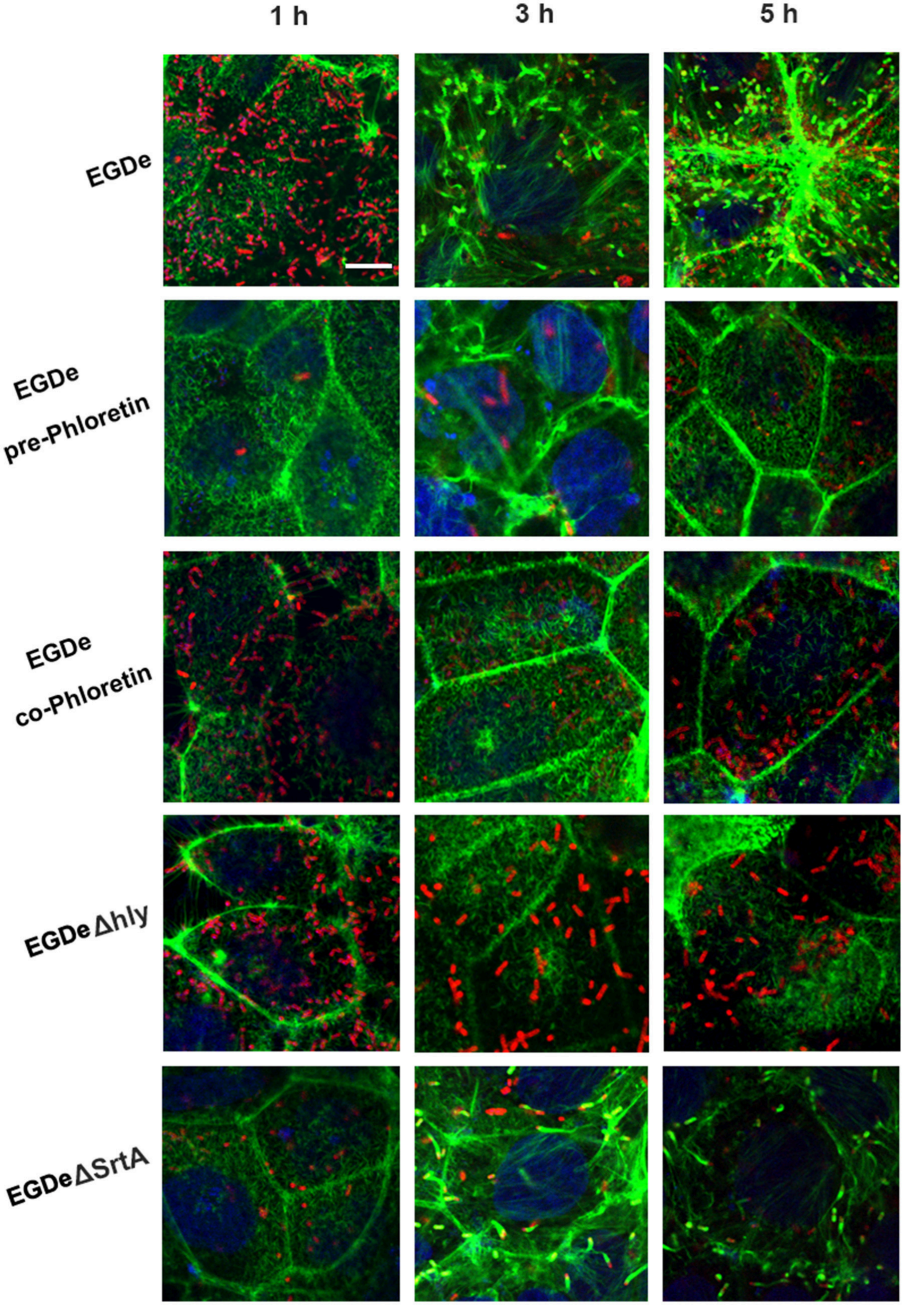
**Phloretin simultaneously inhibits ***L. monocytogenes*** invasion into host cells and escape from internalization vacuoles**. *L. monocytogenes* pre-cultured with or without phloretin (58.34 μM) were co-incubated with host cells at an MOI of 100 in the presence or absence of phloretin (58.34 μM). At 1, 3, and 5 h post-infection, F-actin in the host cells and bacteria was stained with Alexa Fluor 488-conjugated phalloidin antibodies (green) and Alexa Fluor 594-conjugated antibodies (red), respectively; nuclei were stained blue with DAPI. Infection with EGDe cells showed invasion of bacteria into host cells and bacterial escape from vacuoles, as indicated by the numerous bacteria (red) observed in the cells at 1 h post-infection and the fact that most bacteria (red) were surrounded by actin (green) at 5 h post-infection. Significant defects in host cell invasion and bacterial escape from vacuoles were observed for the samples infected with EGDeΔ*srtA* or EGDeΔ*hly* cells. Note that phloretin treatment remarkably inhibited the escape of EGDe cells from vacuoles into cytoplasm, indicated by the fact that the majority of bacteria were not surrounded by F-actin. Importantly, significantly reduced bacterial invasion was observed for the samples infected with EGDe cells pre-treated with phloretin, but not samples infected with EGDe cells without phloretin pre-treatment. Scale bar, 10 μm.

Cell-surface proteins containing the peptide motif LPXTG are linked to cell walls by SrtA and facilitate *L. monocytogenes* invasion into host cells (Li et al., 2016). Following internalization, secreted LLO facilitates *L. monocytogenes* escape from internalization vacuoles into cytoplasm by lysing biomembranes (Hamon et al., 2012). The *in vitro* inhibition of SrtA catalytic activity and LLO expression by phloretin led us to analyze the effects of phloretin treatment on *L. monocytogenes* invasion into host cells and escape from vacuoles. A CFU assay was employed to measure the total number of bacteria adhered to and invaded into host cells at 1 h post-infection. In line with our earlier research, phloretin pre-treated EGDe bacteria showed a significantly diminished ability to invade cells, but this inhibition was not observed for cells that did not receive the pre-treatment (Figure 4A). Additionally, no difference was observed for the samples infected with EGDeΔ*srtA* co-treated or pre-treated with phloretin, suggesting that the inhibition of phloretin against bacterial entry by targeting SrtA. Immunostaining was used to locate extracellular or endocellular bacteria. Extracellular *L. monocytogenes* were stained in red, while total *L. monocytogenes* (intracellular + extracellular) were stained in green. Consistent with our previous study, the *L. monocytogenes* EGDe strain could effectively internalize into Caco-2 cells when co-cultured with host cells; however, the internalization was significantly defective for strain EGDeΔ*srtA*. Pre-treatment with phloretin significantly reduced the entry of EGDe bacteria into host cells (Figure 4B). To directly observe the details of this inhibition, F-actin in host cells and bacteria were stained with Alexa Fluor 488-conjugated phalloidin antibodies (green) and Alexa594-conjugated antibodies (red), respectively. DAPI was used for nuclear staining. In line with the above results, bacterial entry into host cells was reduced only for the samples infected with EGDe cells pre-exposed to phloretin (Figure 5). At 3 h or 5 h post-infection, in contrast to the findings for the EGDeΔ*hly* bacteria, most of the EGDe bacteria colocalized with F-actin, indicative of their cytosolic localization (Figure 5). Although the entry of EGDeΔ*SrtA* bacteria was substantially reduced, their escape from vacuoles into cytoplasm appeared similar to the EGDe bacteria. Importantly, bacterial escape was significantly decreased in the cells infected with the EGDe strain, regardless of whether pre-incubation with phloretin occurred (Figure 5). Taken as a whole, our data indicate that pre-treatment of EGDe cells with phloretin simultaneously inhibits *L. monocytogenes* invasion into Caco-2 cells and cell escape from internalization vacuoles.

### Phloretin reduces cytosolic *L. monocytogenes* multiplication and bacteria-induced cytotoxicity

**Figure 6:**
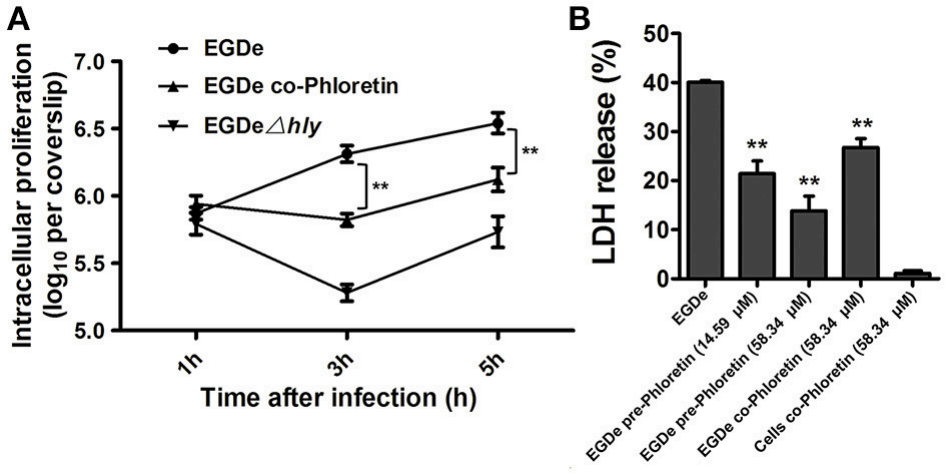
**Inhibition of cytosolic ***L. monocytogenes*** multiplication and bacteria-induced cytotoxicity by phloretin. (A)** Caco-2 cells were infected with *L. monocytogenes* in the presence or absence of phloretin (58.34 μM). After 1, 3, and 5 h of infection, cells were lysed with 0.2% Triton X-100, and CFUs were counted on coverslips to determine the intracellular growth rates of the bacteria. **(B)** Cells were infected with phloretin-pre-treated *L. monocytogenes* at the indicated concentrations or non-pre-treated *L. monocytogenes* for 5 h, and LDH release was detected using a Cytotoxicity Detection Kit to evaluate the cytotoxicity induced by *L. monocytogenes*. ^*^P < 0.05; ^**^P < 0.01.

The numbers of viable bacteria present in the cytoplasms of an infected cell monolayer with or without phloretin treatment were determined with CFU assays at 1, 3, and 5 h after infection. The wild type EGDe strain showed uninhibited growth in cytoplasm, while the endocellular growth of the EGDeΔ*hly* strain was obviously suppressed (Figure 6A). Importantly, the addition of phloretin during infection significantly reduced the multiplication of intracellular bacteria (Figure 6A). The release of LDH into culture supernatant is an indicator of cell death. All the phloretin-treated groups showed decreased LDH release compared with the control group that was infected with EGDe cells but not treated with phloretin (Figure 6B), indicating that cell death was reduced in the presence of phloretin. This inhibitory effect was much more pronounced in the sample infected with bacteria pre-treated with phloretin compared to the sample infected with bacteria without the pre-treatment (Figure 6B). Furthermore, pre-treatment with 14.59 μM phloretin provided greater protection against cell death mediated by the EGDe strain compared to treatment with 58.34 μM phloretin during infection (Figure 6B). Additionally, phloretin treatment did not significantly induce cytotoxicity in Caco-2 cells. Taken together, our results show that phloretin reduces cytosolic *L. monocytogenes* multiplication and bacteria-induced cytotoxicity by simultaneously targeting LLO and SrtA.

### Phloretin protects mice from *L. monocytogenes* infection

**Figure 7:**
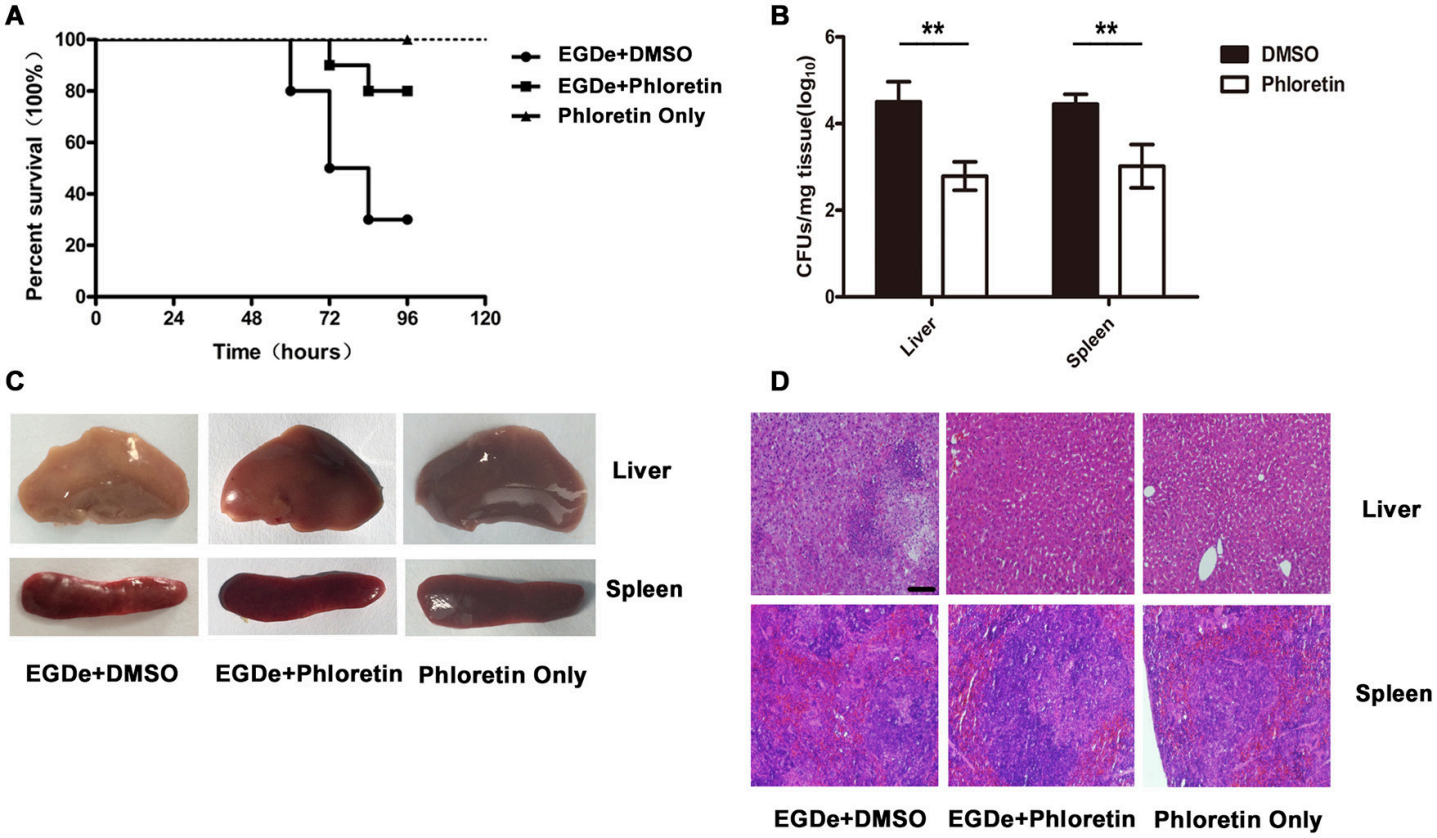
**Phloretin protects mice from ***L. monocytogenes*** infection**. Mice were intraperitoneally infected with *L. monocytogenes* and treated with phloretin or DMSO as a control. The mice were sacrificed 48 h post-infection to determine the bacterial burden and analyze the pathological damage resulting from infection. **(A)** Survival analysis of infected or uninfected mice treated with phloretin over a 96-h period. **(B)** Bacterial burdens in the livers and spleens of the infected mice. Treatment with phloretin alleviated the gross pathological changes **(C)** and histopathological changes **(D)** observed in the livers and spleens of the infected mice. Scale bar, 50 μm. ^*^P < 0.05; ^**^P < 0.01.

Mutants lacking SrtA or LLO exhibited remarkably decreased virulence, as shown in our previous work, in a mouse model of *L. monocytogenes* infection (Wang et al., 2015a; Li et al., 2016). Here, the observed phloretin-mediated inhibition against *L. monocytogenes* invasion into host cells and escape from internalization vacuoles into cytoplasm prompted us to further evaluate the therapeutic effects of this compound for *L. monocytogenes* infection in a mouse model. Consistent with previous studies, 75% of infected mice had died within 96 h after intraperitoneal injection of 2 × 10^7^ CFUs of bacteria per mouse, with a median survival time of 78 h (Figure 7A). However, only 25% of the infected mice that were treated with phloretin had died by 96 h post-infection (Figure 7A). The infected mice that had received phloretin exhibited a significantly higher survival rate than the control group treated with DMSO (Figure 7A). Furthermore, the mice treated only with phloretin and not subjected to bacterial infection all survived, indicating that phloretin itself has no toxicity to mice (Figure 7A). CFUs on TSB agar plates were counted to measure the bacterial burden present in liver or spleen tissues isolated from infected mice treated with or without phloretin. As expected, the numbers of viable bacteria colonized in the livers and spleens of the phloretin-treated mice were significantly lower than those from the infected mice treated with DMSO (Figure 7B). Gross inspection indicated that the liver tissues of the infected mice treated with DMSO became gray in appearance with white necrotic foci, while the spleen tissue from this group became dusky-red in color and had a tight texture (Figure 7C). In contrast, the pathological changes in the infected mice that received phloretin were clearly ameliorated (Figure 7C). Furthermore, no pathological changes were observed in the uninfected mice treated with phloretin, further suggesting that phloretin has no toxicity for mice (Figure 7C). In agreement with these findings, evaluation of tissue sections showed significant accumulations of inflammatory cells (dark blue or purple) in the infected mice treated with DMSO (Figure 7D). However, only minor inflammatory lesions were found in the phloretin-treated mice infected with *L. monocytogenes*, and no gross lesions or pathological changes were observed in the uninfected mice that received phloretin (Figures 7C,D). Taken together, our results show that phloretin treatment provides systemic protection against *L. monocytogenes* infection in mice.

## Discussion

Many surface proteins in most Gram-positive bacteria are covalently linked to the cell wall by the transpeptidase SrtA (Spirig et al., 2011). These proteins are crucial for the adherence, invasion and colonization of bacteria during infection, especially for the intracellular bacteria *L. monocytogenes* (Mariscotti et al., 2012; Li et al., 2016). Following internalization into host cells, the pore-forming toxin LLO, one of the members of the cholesterol-dependent cytolysin (CDC) family, breaches the phagosomal membranes of host cells, facilitating the escape of *L. monocytogenes* entrapped in internalization vacuoles into cytoplasm without host immune recognition (Hamon et al., 2012). Knockout of SrtA or LLO from *L. monocytogenes* significantly affects bacterial internalization (Bierne et al., 2002) and vacuole escape (Hamon et al., 2012), respectively. Furthermore, the virulence of these mutant strains was remarkably decreased in a mouse model (Bierne et al., 2002; Lety et al., 2002), indicating that both SrtA and LLO are potential anti-virulence drug targets for combatting *L. monocytogenes* infection. In our previous studies, treatment with chalcone (Li et al., 2016), an inhibitor of SrtA, or fisetin (Wang et al., 2015a), an inhibitor of LLO, provided robust protection against *L. monocytogenes* infection *in vitro* and *in vivo*. However, the therapeutic effects of chalcone and fisetin for *L. monocytogenes* infection are unsatisfactory due to only one virulence factor targeted by the natural compounds. Thus, we reasoned that an agent that could simultaneously target both virulence factors would produce better results.

To validate this hypothesis, phloretin, a natural polyphenolic compound derived from apples and pears (Crespy et al., 2001) with little anti-*L. monocytogenes* activity, was identified as an effective inhibitor of *L. monocytogenes* that could simultaneously target SrtA and LLO according to a SrtA catalytic activity inhibition assay and a hemolysis assay. SrtA catalytic activity and LLO expression in bacterial culture supernatant and cell lysate were both significantly inhibited following treatment with 58.34 μM phloretin. However, the phloretin treatment did not affect the transcription of *hly*, indicating that the decreased LLO expression may have resulted from inhibition of LLO translation in cytoplasm. Additionally, the half maximal inhibitory concentration (IC_50_) of phloretin against SrtA was measured as 37.24 μM. Furthermore, the growth of *L. monocytogenes* was not visibly affected by the presence of phloretin at the concentration required for such inhibition. These data indicate that phloretin may represent an effective anti-virulence agent that works by simultaneously targeting SrtA and LLO without putting selective pressure on *L. monocytogenes*. Consistent with this idea, the invasion of *L. monocytogenes* into Caco-2 cells was significantly blocked after an overnight incubation with phloretin, however, this inhibition was not observed for the samples without phloretin treatment. Furthermore, treatment with phloretin attenuated the escape of *L. monocytogenes* into cytoplasm, leading to degradation of the bacteria by host cells. Although the cytotoxicity induced by *L. monocytogenes* was attenuated in the presence of either a SrtA inhibitor or an LLO inhibitor, pre-incubation of bacteria with phloretin overnight provided more effective protection against *L. monocytogenes*-mediated cytotoxicity than no pre-incubation. This difference was also observed between samples pre-incubated with a lower concentration of phloretin (14.59 μM) and those co-incubated with a higher concentration of phloretin (58.34 μM). Therefore, phloretin inhibited *L. monocytogenes* invasion and escape from vacuoles by simultaneously targeting SrtA and LLO. More importantly, phloretin treatment provided robust protection against *L. monocytogenes* infection in a mouse model. To the best of our knowledge, this is the first study to demonstrate that an inhibitor that simultaneously targets these two virulence factors can be an effective anti-infective against *L. monocytogenes*.

To further characterize the mechanism by which phloretin inhibits SrtA activity, a MD simulation of SrtA-phloretin binding was performed. The binding of phloretin to a specific binding pocket on SrtA critically affected the binding of SrtA to its substrate. We thus analyzed the potential conformational changes in SrtA upon binding to phloretin using a MD-simulated trajectory. In the free protein, the distances between Ala53-Ile114 and His55-Arg125 (in the active pocket of SrtA) ranged from 0.42 to 0.58 nm and 0.79 to 1.02 nm over the time course of the simulation, with average distances of 0.54 and 0.88 nm (Figures 3D,E). Notably, in the SrtA-phloretin complex, these distances ranged from 0.45 to 0.64 nm and 0.92 to 1.44 nm, with average distances of 0.59 and 1.19 nm (Figures 3D,E). The changes in the distances between Ala53-Ile114 and His55-Arg125 upon SrtA's interaction with phloretin suggest that the active pocket of SrtA undergoes a significant conformational change upon engagement with phloretin (Figures 3D,E). As a result, the active pocket can no longer interact with the normal substrate of SrtA, thereby reducing the biological activity of SrtA.

Both SrtA and LLO are indispensable virulence determinants for *L. monocytogenes* virulence. Thus, the double inhibition of SrtA and LLO caused by phloretin may offer a more effective anti-virulence strategy for infections with *L. monocytogenes*.

## Author contributions

Conceived and designed the experiments: XD, YY, and JW. Performed the experiments: JW, BL, ZT, XZ, XW, and XN. Analyzed the data: BZ and GL. Wrote the paper: XD and JW.

## Funding

This work was supported by the National Nature Science Foundation of China (grant 31130053) and the National Basic Research Program of China (grant 2013CB127205).

### Conflict of interest statement

The authors declare that the research was conducted in the absence of any commercial or financial relationships that could be construed as a potential conflict of interest.
